# Dr. William C. L. Meisburger

**Published:** 1907-01

**Authors:** 


### OBITUARY.

DR. WILLIAM C. L. MEISBURGER, of Buffalo, died December 8, 1906, aged forty-one years. He was the son of the late Dr. William Meisburger, who was a well known practising physician in Buffalo for many years.

Dr. Meisburger, the son, graduated from the medical department of the University of Buffalo in 1887, and afterward served two years as house surgeon at the Buffalo General Hospital. He was then appointed first assistant physician at the Wisconsin State Hospital for the insane at Wawautosa, where he remained for four years. He then returned to Buffalo, where he practised until failing health a year or two ago caused his retirement. He was a member of the Medical Society of the County of Erie and of the Roswell Park Medical Club. He is survived by his wife and a brother, Dr. Louis Meisburger, who is a dentist.

